# Giant Topological Hall Effect in the Noncollinear Phase of Two-Dimensional Antiferromagnetic Topological Insulator MnBi_4_Te_7_

**DOI:** 10.1021/acs.chemmater.1c02625

**Published:** 2021-10-19

**Authors:** Subhajit Roychowdhury, Sukriti Singh, Satya N. Guin, Nitesh Kumar, Tirthankar Chakraborty, Walter Schnelle, Horst Borrmann, Chandra Shekhar, Claudia Felser

**Affiliations:** Max Planck Institute for Chemical Physics of Solids, 01187 Dresden, Germany

## Abstract

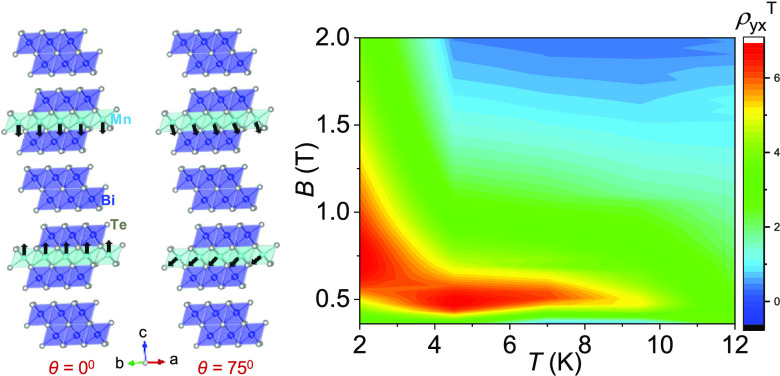

Magnetic topological insulators provide an important platform for realizing several exotic quantum phenomena, such as the axion insulating state and the quantum anomalous Hall effect, owing to the interplay between topology and magnetism. MnBi_4_Te_7_ is a two-dimensional Z_2_ antiferromagnetic (AFM) topological insulator with a Néel temperature of ∼13 K. In AFM materials, the topological Hall effect (THE) is observed owing to the existence of nontrivial spin structures. A material with noncollinearity that develops in the AFM phase rather than at the onset of the AFM order is particularly important. In this study, we observed that such an unanticipated THE starts to develop in a MnBi_4_Te_7_ single crystal when the magnetic field is rotated away from the easy axis (*c*-axis) of the system. Furthermore, the THE resistivity reaches a giant value of ∼7 μΩ-cm at 2 K when the angle between the magnetic field and the *c*-axis is 75°. This value is significantly higher than the values for previously reported systems with noncoplanar structures. The THE can be ascribed to the noncoplanar spin structure resulting from the canted state during the spin-flip transition in the ground AFM state of MnBi_4_Te_7_. The large THE at a relatively low applied field makes the MnBi_4_Te_7_ system a potential candidate for spintronic applications.

## 1 Introduction

Magnetic topological insulators (MTIs) have drawn significant attention owing to the interplay between the magnetic order and nontrivial band topology, which provides an important platform for realizing emergent quantum phenomena such as the axion insulating state, the quantum anomalous Hall effect (QAHE), the topological magnetoelectric effect, and Majorana modes.^1−5^ Interestingly, the Hall effect in MTIs exhibits a unique behavior compared to their nonmagnetic counterparts owing to nontrivial spin arrangements.^5^ In ferromagnetic (FM) compounds, there is an additional contribution to the Hall effect, which is known as the anomalous Hall effect (AHE) and occurs in a zero magnetic field.^6−9^ The AHE originates from two qualitative mechanisms. The first is an extrinsic contribution, which involves skew scattering and side-jump scattering. The second is an intrinsic contribution arising from magnetization and spin–orbit interaction, which is further related to inverse space Berry curvature (BC).^7,10,11^

In the last few decades, a vast number of topological phenomena have been explained using BC.^4,5,12^ Thus, it is always interesting to study the effect of BC on the electronic properties of topological quantum materials. In an inverse space, the BC of topological bands leads to the intrinsic part of the AHE.^4,9,12^ In contrast, in a real space, a noncoplanar spin texture with nonzero scalar spin chirality, such as skyrmions, acts as a magnetic field and produces an additional Hall signal in a system. This is known as the topological Hall effect (THE).^13,14^ Moreover, the presence of noncoplanar spin structures without skyrmions in a lattice generates a TH signal in a system.^7,15−17^ In this scenario, the total Hall resistivity, ρ*_yx_*, is the sum of three contributions: ρ*_yx_* = *R*_0_*B* + *R*_S_*M* + ρ*_yx_*^T^. The first, second, and third terms denote the ordinary, anomalous, and topological Hall resistivities, respectively. *R*_0_ and *R*_S_ represent the ordinary and anomalous Hall coefficients, respectively.^18,19^

The THE is commonly considered to be a feature of topologically nontrivial spin textures, particularly for magnetic skyrmions, which have potential applications as memory and logic elements in future computing devices.^14,19,20^ Concerning experiments, probing the THE *via* magnetotransport measurements has the advantage of providing information about a system without the requirement of a low-temperature Lorentz transmission electron microscope or neutron diffraction study.^19,21^

The THE was first observed in the skyrmionic phases of MnSi^22^ and FeGe^23^ (non-centrosymmetric cubic structure), with an extremely small topological Hall resistivity (10^–3^–10^–2^ μΩ-cm). The THE has been widely studied in several systems with noncoplanar antiferromagnetic (AFM) spin structures such as MnP,^24^ Mn_5_Si_3_,^15^ and YMn_6_Sn_6_,^16^ kagome lattices such as Mn_3_Sn^25^ and Fe_3_Sn_2_,^26^ and frustrated magnets such as PdCrO_2_,^27^ Pr_2_Ir_2_O_7_,^28^ and Gd_2_PdSi_3_.^19^

Recently, there has been a lot of focus in realizing two-dimensional (2D) topological semimetals, TIs, and discovering their new chemistry.^29,30^ It is possible to identify exciting new materials that are easy to synthesize, air stable, and cost-effective with chemical intuition.^29^ In this context, solid-state chemistry can make a significant contribution to the discovery of new quantum states by providing a better understanding of the structure–property relationship. In recent years, materials from the homologous series of the MnBi_2*n*_Te_3*n*+1_ family have attracted attention in the field of two-dimensional AFM topological insulators. MnBi_2_Te_4_ (*n* = 1) has been identified as the first intrinsic van der Waals (vdW) antiferromagnet with a nontrivial topological surface state.^31,32^ Its crystal structure is similar to that of Bi_2_Te_3_, which is a well-known topological insulator. The next member from the MnBi_2*n*_Te_3*n*+1_ family, *i.e.*, MnBi_4_Te_7_ (*n* = 2), crystallizes in a trigonal structure with the *P*3̅*m*1 space group.^33,34^ It is worth noting that the advantages of the materials from this homologous series are (i) periodical crystalline structure, (ii) intrinsic magnetism, and (iii) van der Waals gap, which guarantee that the chemical potential changes gradually at the interface.^35^ First-principles density functional theory calculation predicts that it is a  AFM topological insulator and a possible candidate for realizing the axion insulating state.^33^ It differs from MnBi_2_Te_4_ in two aspects. First, a nonmagnetic layer (Bi_2_Te_3_) separates the magnetic septuple layers (SLs), thereby reducing interlayer coupling. Second, the surface termination (magnetic or nonmagnetic) is expected to be different.

Unlike MnBi_2_Te_4_, a previous magnetotransport research on MnBi_4_Te_7_ showed that it features a direct spin-flip transition from the AFM phase to the FM phase at a low magnetic field (∼0.2 T), without any canted AFM phase.^32−34^ However, it will be interesting to explore if MnBi_4_Te_7_, which also belongs to the homologous family of MnBi_2*n*_Te_3*n*+1_, also exhibits a noncoplanar spin structure under certain conditions. This has not been studied till now. Because of the weak AFM interaction in MnBi_4_Te_7_, it is a common intuition to tailor its spin structure from the original one and study the effect by simple magnetic measurement. Here, the noncoplanar spin structure will govern the magnetism of the system. Thus, Hall effect measurements enable the solid-state chemists to get a much deeper insight into the spin structure of 2D materials. Such an understanding might be helpful to design phase diagrams of magnetic ground states in other novel 2D TI families, which was not observed earlier.

In this work, we investigate the angular variation in the Hall effect in a two-dimensional van der Waals AFM topological insulator, *i.e.*, a MnBi_4_Te_7_ single crystal. We observed an unexpected THE when the magnetic field was rotated away from the easy axis (*c*-axis) of the system. A large THE resistivity of ∼7 μΩ-cm was observed at 2 K and θ = 75° with respect to the *c*-axis. This THE resistivity decreased as temperature increased. In this measurement configuration, the ground AFM state of MnBi_4_Te_7_ experienced a canted state from the spin-flip transition. This resulted in a noncoplanar spin structure, which was the origin of the observed THE. The observed value of the THE resistivity was significantly higher than the previously reported values for noncoplanar magnetic structures. Our finding highlights the importance of the previously unexplored noncoplanar structure in a two-dimensional system to enhance the understanding of the THE.

## 2 Experimental Section

### 2.1 Single-Crystal Growth of MnBi_4_Te_7_ and Characterizations

The Bi_2_Te_3_ flux procedure was used to grow the single crystals of MnBi_4_Te_7_. As-purchased high-quality elemental manganese (99.999%, Alfa Aesar), bismuth (99.997%, Alfa Aesar), and tellurium (99.9999%, Alfa Aesar) were mixed in a molar ratio of Mn:Bi:Te of 1:10:16. All of the elements were loaded into an alumina crucible, which was vacuum-sealed in a quartz tube under 10^–5^ Torr. The tube was heated to 1233 K for 12 h, and then submerged for 24 h before being progressively cooled to 855 K for 100 h. After centrifuging at 855 K to remove excess Bi_2_Te_3_, the crystals were recovered. The single crystal has a typical dimension of 2 × 2 × 0.3 mm^3^. A Huber image plate Guinier G670 camera operated with CuK_α1_ radiation (λ = 1.54056 Å) was used to measure powder X-ray diffraction (PXRD) at room temperature. Figure S1 shows the powder XRD data for the crushed crystal. White-beam backscattering Laue X-ray diffraction was used to determine the single crystallinity of the as-grown crystal. On a single crystal diffractometer, the quality and orientation of the as-grown crystals were assessed using transmission of thin edges. Unambiguous indexing revealed an expected trigonal unit cell with lattice parameters *a* = 4.37 Å and *c* = 23.80 Å. Oscillation images confirmed the determined unit cell and symmetry as well as good crystal quality (Figure S2). Scanning electron microscopy along with an energy-dispersive EDAX analyzer was used to evaluate the composition of the MnBi_4_Te_7_ crystal.

### 2.2 Magnetization Measurements

An MPMS3 instrument was used for the magnetization measurement.

### 2.3 Electrical Transport Measurements

A physical property measurement system (PPMS9) (ETO option, Quantum Design) was employed to measure the electrical transport. For transport studies, the sample was cut into a standard rectangular shape and a six-probe technique was used to simultaneously measure the normal resistivity and Hall resistivity. The final resistivity and (Hall) data were symmetrized (antisymmetrized) to eliminate the misalignment of the electrodes.

## 3 Results and Discussion

**Figure 1:**
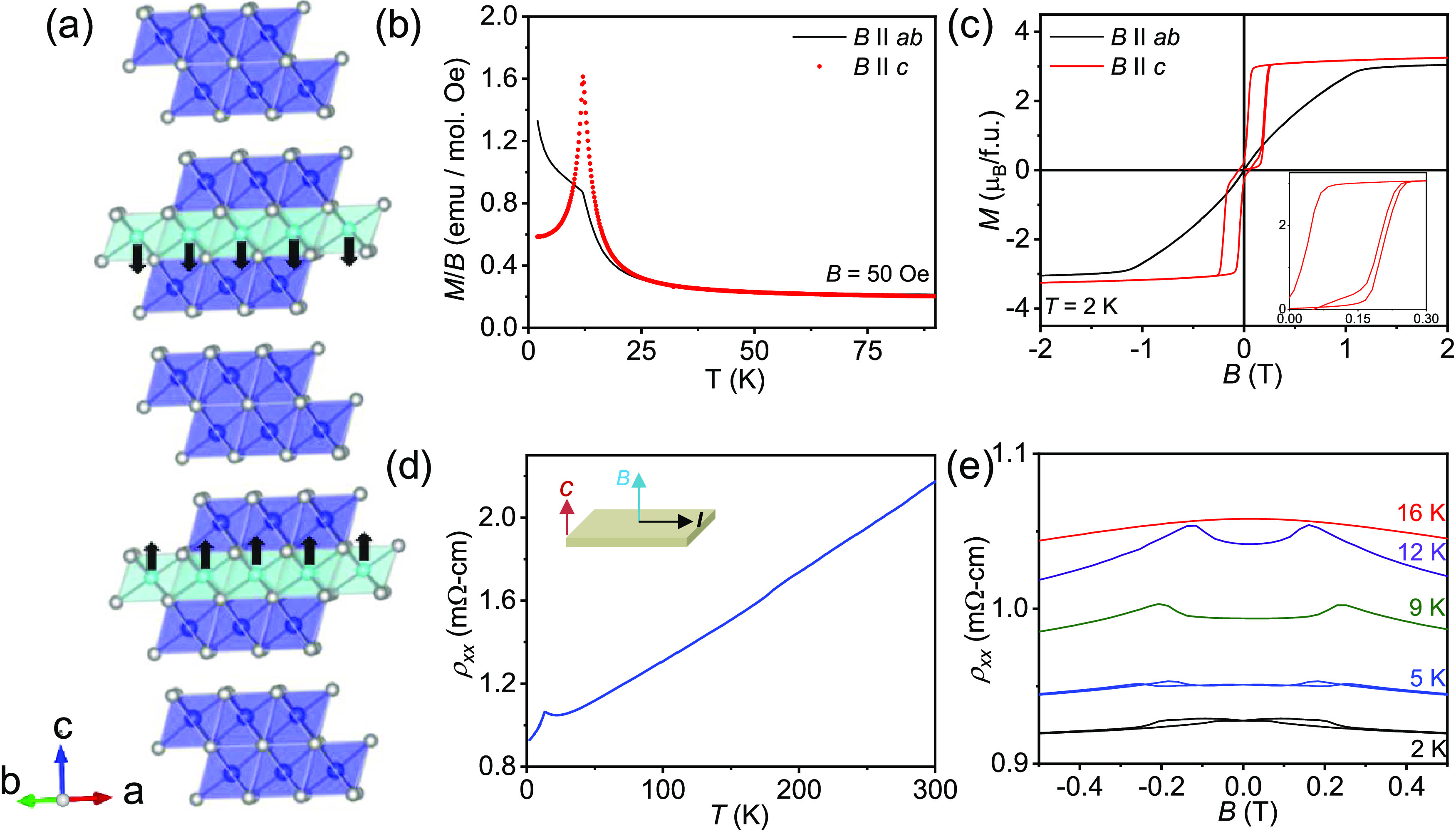
(a) Crystal structure of MnBi_4_Te_7_ (sky blue, dark blue, and white atoms represent Mn, Bi, and Te, respectively). (b) Temperature-dependent field-cooled magnetic susceptibility at *B* = 50 Oe for *B* || *ab* and *B* || *c*. (c) Isothermal magnetization at 2 K for *B* || *ab* and *B* || *c*. The inset shows the virgin line for *B* || *c*. (d) Variation in resistivity, ρ_*xx*_, with temperature. (e) Field-dependent resistivity of MnBi_4_Te_7_ at various temperatures.

We have synthesized high-quality single crystals of MnBi_4_Te_7_ from the homologous series MnBi_2*n*_Te_3*n*+1_*via* a Bi_2_Te_3_ flux method (see the Experimental section). MnBi_4_Te_7_ crystallizes in a trigonal structure with the *P*3*®m*1 space group. The structure is characterized by alternate stacking of septuple layers (SL) of MnBi_2_Te_4_ (Te-Bi-Te-Mn-Te-Bi-Te) and quintuple layers of Bi_2_Te_3_ (Te-Bi-Te-Bi-Te) along the *c*-axis via van der Waals interaction, making it an ideal two-dimensional material (Figure 1a). The *d*-orbitals of Mn^2+^ ions, which form long-range FM ordering within the SL, are responsible for the local magnetic moment. In contrast, along the *c*-axis, the magnetic moments from adjacent SLs are antiferromagnetically ordered, forming an A-type AFM state similar to MnBi_2_Te_4_. However, the SLs are separated by a nonmagnetic layer (Bi_2_Te_3_), which reduces the interlayer AFM exchange coupling. This results in a lower Néel temperature (*T*_N_) of ∼13 K for MnBi_4_Te_7_ (Figure 1b) compared to MnBi_2_Te_4_ (∼25 K).^32,33,36^ Selected oscillation images around the main axes of MnBi_4_Te_7_ single crystals are shown in Figure S2, Supporting Information (SI).

The measured magnetic susceptibility and resistivity of MnBi_4_Te_7_ are shown in Figure 1b–e. The longitudinal resistivity, ρ_*xx*_, decreases linearly with temperature up to ∼20 K. As temperature decreases further, ρ_*xx*_ slightly increases and then decreases because of the increase in scattering caused by the fluctuation of magnetic spins as the Néel temperature is reached. This effect is known to occur in low-dimensional magnetic systems (Figure 1d). The abrupt decrease in ρ*_xx_* indicates that local Mn moments form a long-range ordered state at *T* < 13 K; this is consistent with the magnetic susceptibility measurement. We also measured out-of-plane resistance data, and the results are shown in Figure S5b, SI which clearly shows the large transport anisotropy in the system due to the vdW nature of MnBi_4_Te_7_. Figure 1b represents the field-cooled magnetic susceptibility curves on *B* || *ab* (χ_*ab*_) and *B* || *c* (χ_*c*_) planes at a magnetic field of 50 Oe. Figure 1b shows that χ_*c*_ is two orders of magnitude more than that χ_*ab*_, implying that MnBi_4_Te_7_ has significant magnetic anisotropy. The data for the magnetic field applied along the *c*-axis (χ_*c*_) show a peak at ∼13 K, which has been observed for other layered antiferromagnets from the MnBi_2*n*_Te_3*n*+1_ series, such as MnBi_2_Te_4_ (∼25 K) and MnBi_6_Te_10_ (∼11 K).^37,38^

The magnetization isotherm data with on the *B* || *ab* and *B* || *c* at temperatures of 2–30 K are shown in Figures 1c and S4 (SI). It can be clearly seen that MnBi_4_Te_7_ undergoes a first-order spin-flip transition with hysteresis at 2 K (Figure 1c). The hysteresis begins at a low field of ∼0.15 T, rapidly enters the forced FM state, and becomes saturated at 0.22 T. Thus, the magnetization trend of MnBi_4_Te_7_ (*B* || *c*) is strongly different from that of MnBi_2_Te_4_, in which a spin-flop transition occurs at 3.5 T and a transition from a canted AFM phase to an FM phase occurs at ∼8 T. This confirms the weaker interlayer AFM exchange coupling in MnBi_4_Te_7_ compared to MnBi_2_Te_4_.^38^ However, magnetization along the *B* || *ab*-plane requires a high saturation field of ∼1 T, indicating that the *c*-axis is the easy magnetic axis. The observed saturation magnetic moment for Mn is 3.74 μ_B_ at 7 T, which is lower than the theoretical value for *d*^5^ Mn^2+^ (4.6 μ_B_).^33^ The discrepancy between the calculated and observed values mainly arises from the Mn disorders in the synthesized samples.^35^

**Figure 2:**
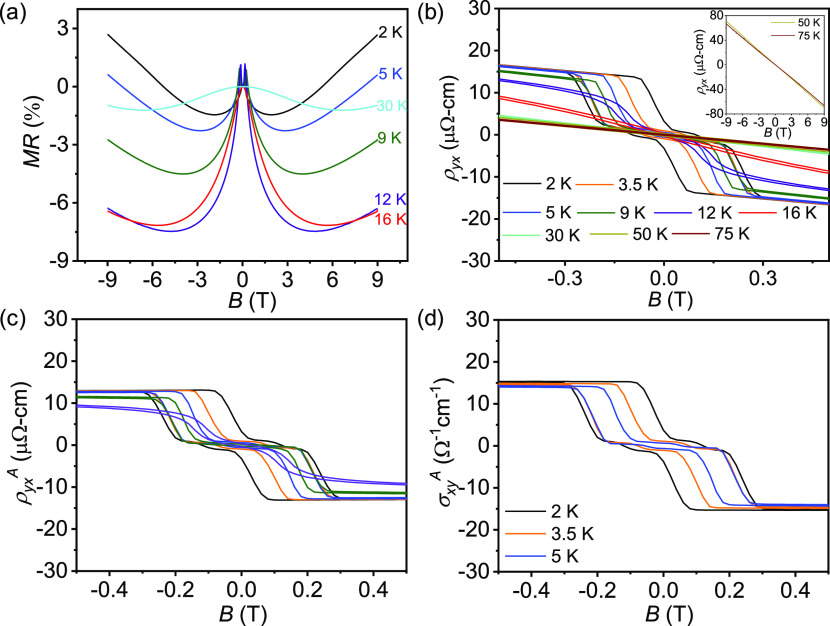
(a) Transverse magnetoresistance at different temperatures. (b) Field-dependent Hall resistivity, ρ*_yx_* (inset: linear field dependence of ρ*_yx_* up to 9 T at *T* > 50 K), (c) anomalous Hall resistivity, ρ*_yx_*^A^, and (d) anomalous Hall conductivity, σ_xy_^A^, at different temperatures.

We measured the longitudinal resistivity and Hall resistivity of the MnBi_4_Te_7_ single crystal. The AFM–FM spin-flip transitions can be clearly seen from the ρ_xx_–*B* plot, where the magnetic field is applied along the *c*-axis and *I* || *ab*-plane (Figure 1e). From the field dependent measured ρ*_xx_*, we calculated the transverse magnetoresistance (MR = (ρ*_xx_*(*B*)-ρ*_xx_*(0)/ρ*_xx_*(0))), and the results are shown in Figure 2a. A maximum negative MR of ∼ 8% is observed at 12 K, which is close to the Néel temperature. The negative MR can be attributed to the suppression of spin-disorder-related scattering, which is generally observed in magnetic systems.^33,34^

The variation in the Hall resistivity with the magnetic field at various temperatures is presented in Figure 2b–d. Figure 2b,c clearly shows that the AHE is present in the system when *T* < *T*_N_, owing to the AFM–FM spin-flip transition; this is consistent with the isothermal magnetization and magnetoresistivity measurements (Figure 1c,e). Thus, the Hall resistivity can be expressed as ρ*_yx_* = *R*_0_μ_0_*H* + *R*_S_*M*. Typically, the anomalous Hall conductivity (AHC, σ*_xy_*^A^) at 2 K is ∼15 Ω^−1^ cm^−1^, which is consistent with the previous reports.^33,34^ Wu et al. proposed that the AHC in the present system has a dominant contribution from BC.^34^ At a high temperature of *T* > 50 K, the Hall resistivity shows a linear field dependence up to 9 T, suggesting a single carrier band in MnBi_4_Te_7_. The electron carrier density of our sample is ∼8 × 10^19^ cm^–3^ at 50 K.

**Figure 3:**
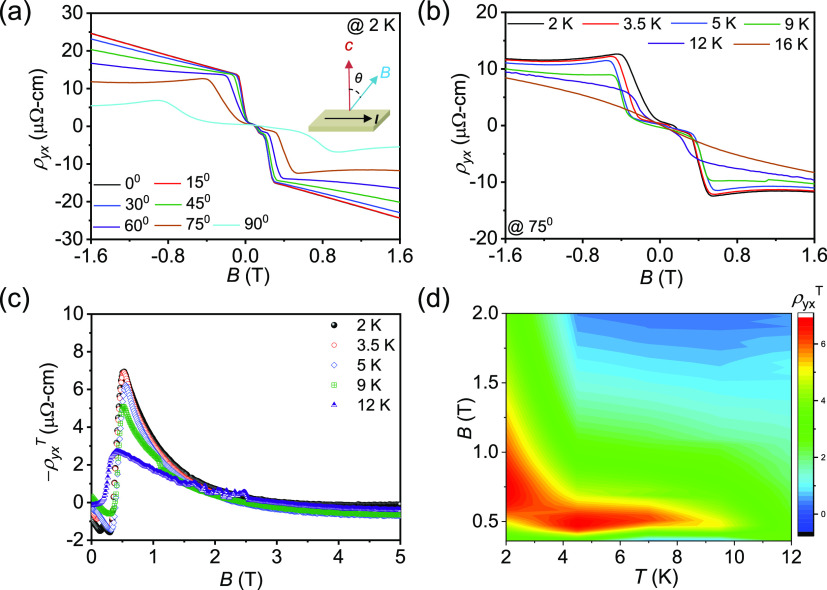
(a) Field-dependent Hall resistivity, ρ*_yx_*, at different values of θ (inset represents measurement setup). (b) Field-dependent Hall resistivity, ρ*_yx_*, and (c) topological Hall resistivity, ρ*_yx_*^T^, at θ = 75° at various temperatures. (d) Contour plot of topological Hall resistivity, ρ*_yx_*^T^, as a function of temperature and the magnetic field.

We did not observe any evidence of the existence of a noncoplanar structure from the magnetotransport data in the geometry *B* || *c*-axis and *I* || *ab*-plane measurements.^33,34^ As the spins (magnetization) in the *ab*-plane and the *c*-plane have completely different behaviors, it is interesting to investigate the effect of spin fluctuations on the magnetotransport for field directions in between these limits. We investigated ρ*_yx_* and ρ*_xx_* while steadily rotating the magnetic field (*B*) from the *c*-axis to the *ab*-plane. The schematic of our measurement is presented in Figure 3 (inset), where θ represents the angle between *B* and the *c*-axis. At θ = 0°, the MR and Hall resistivity (Figure 2) are consistent with the earlier report.^33,34^ Below *T*_N_, e.g., at 2 K, ρ*_yx_* steadily decreases as θ increases from 0 to 90°. In addition, a hump-like anomaly appears in the low-*B* region (<1 T), which becomes pronounced at θ = 75°, as shown in Figure 3a. We focus on θ = 75° to investigate the transport properties at different temperatures (below and above the Néel temperature) (Figure 3b).

**Figure 4:**
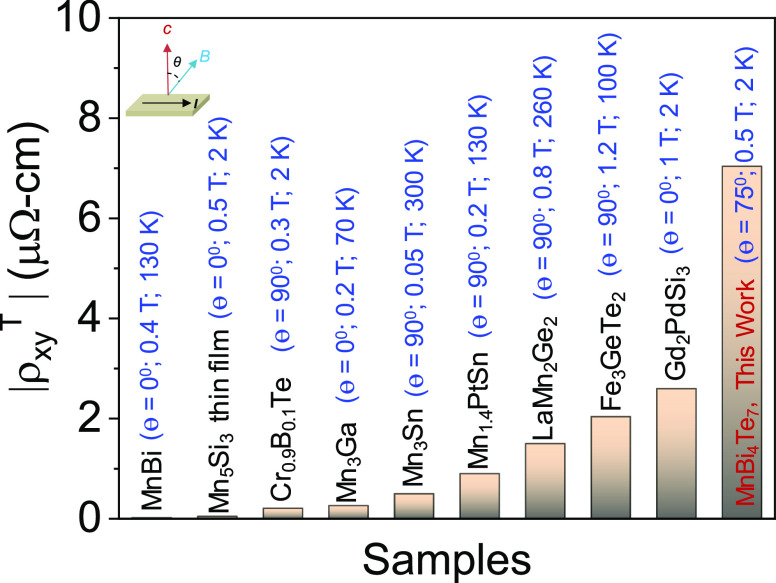
Comparison of maximum topological Hall resistivity obtained in the present work with previously reported values.^15,17,19,39−44^ We have listed the experimental conditions of each material when they reach their maximum topological Hall resistivity.

It should be noted that although we observe a hump-like feature in the low *B* region of the plot of ρ*_yx_*, no such anomaly is observed in the *M* vs *B* curve (at θ = 75°) (Figure S6, SI). This strongly supports the presence of a Hall effect in addition to the ordinary Hall effect and AHE, namely, the THE, which is different from the *B* || *c*-axis measurement (Figure 2). Thus, in this scenario, ρ_yx_ can be expressed as ρ*_yx_* = *R*_0_μ_0_*H* + *R*_S_*M* + ρ*_yx_*^T^. After subtracting the first two terms, we obtain the values of ρ*_yx_*^T^ at different temperatures for θ = 75° (Figure 3c). To clearly observe the variation in the THE, we create a contour plot of the *B*–*T* phase diagram by extracting ρ_xy_^T^ over the measured temperature range (Figure 3d). Surprisingly, a giant topological Hall resistivity (ρ*_yx_*^T^) of ∼ 7 μΩ-cm is observed at 2 K, which is significantly higher than any previously reported value (Figure 4).^15,17,19,39−44^ This makes the MnBi_4_Te_7_ system a potential candidate for spintronic applications.

**Figure 5:**
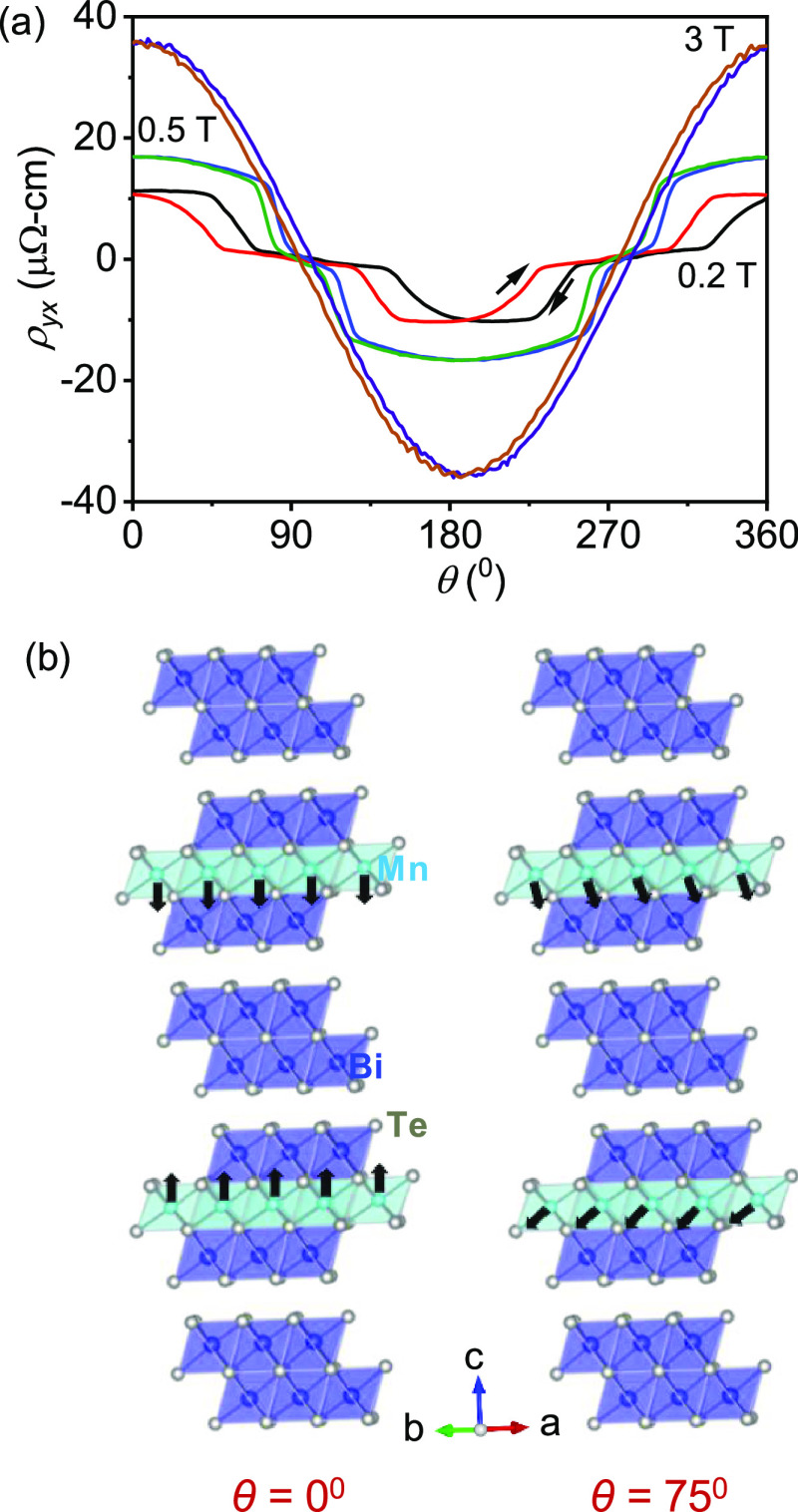
(a) Hall resistivity at 3.5 K as a function of θ at different fields. (b) Schematic of the possible origin of the noncoplanar structure at θ = 75° from θ = 0° (sky blue, dark blue, and white atoms represent Mn, Bi, and Te, respectively).

We have measured ρ*_yx_* as a function of θ at 3.5 K at various magnetic fields (Figure 5a). At θ = 0° and *B* = 0.2 T, ρ*_yx_* is ∼ 10 μΩ-cm. As we rotate the field clockwise from the *c*-axis, ρ*_yx_* initially remains flat and then abruptly decreases to almost zero around θ = 45°. Interestingly, hysteresis is observed between the clockwise and anticlockwise rotation of *B*, indicating that this *B*-direction-sensitive phase change is of the first order. With increasing the field, hysteresis slowly decreases. After the application of 0.7 T, hysteresis vanishes (Figure S8, SI). The critical magnetic field for hysteresis is 0.7 T in the present system. A similar observation was previously reported in a frustrated triangular lattice of a Gd_2_PdSi_3_ system, in which the skyrmionic lattice was limited to only a two-dimensional space.^19^ Thus, the abovementioned behavior indicates the possibility of realizing a skyrmion lattice in the present system, which is composed of the stacked FM triangular-lattice layers of MnBi_2_Te_4_. The amplitude of the topological Hall resistivity abruptly transitions from a finite value to zero, providing a measure of the topological number for the spin texture. Further studies are required to clarify the microscopic origin of the BC in the present system. In contrast, at a higher magnetic field (*B* = 3 T), ρ*_yx_* follows a simple cos θ relation without any hysteresis. In this case, the AHE simply scales with the out-of-plane component of the magnetic field. However, the components of the AHE are expected to become zero at θ = 90 or 270°.

An AFM state with a noncollinear spin structure is likely to have a large THE.^45^ In such materials, symmetry breaking combined with significant spin–orbit coupling can lift spin degeneracy, producing a net Berry curvature in momentum space and an intrinsic AH effect. Earlier Wu et al. proposed that the AHC in MnBi_4_Te_7_ has a dominant contribution from the Berry curvature.^34^ The observed THE can be attributed to the relative strength of interlayer exchange coupling (*J*) and uniaxial anisotropy (*K*), which plays a crucial role in controlling the transition from the A-type AFM state to the FM state.^46^ In MnBi_4_Te_7_, there exists a competition between AFM and FM couplings owing to the separation of the two magnetic SLs of MnBi_2_Te_4_ by a nonmagnetic layer (Bi_2_Te_3_), which reduces the interlayer AFM exchange coupling. Tan et al. recently reported that a metamagnetic phase exists in MnBi_4_Te_7_, which is controlled by uniaxial anisotropy at a low temperature.^46^ When a magnetic field is applied along the *c*-axis, either a parallel or an antiparallel alignment of sublattice magnetizations occurs. This results in two spin-flip transitions in MnBi_4_Te_7_, accompanied by hysteresis. Depending on the relative strength of *K* and *J* (*K*/*J* ratio), the spin-flip transition might change to either a canted state or an FM-like alignment under a finite magnetic field. According to a recent theoretical model, the AFM state of MnBi_4_Te_7_ experiences a canted state from the spin-flip transition at *K*/2*J* < 1/3.^46^ Therefore, the spins of Mn^2+^ become noncoplanar during the spin-flip process, resulting in the THE. Thus, we observe the THE owing to the existence of the canted spin structure in MnBi_4_Te_7_ at *θ* = 75° (Figure 5b). As the magnetic field is increased further, the THE is suppressed because the spins become parallel. The noncollinear spin structure can be ascribed to the significant TH value obtained here in the CAFM phase of MnBi_4_Te_7_. This finding means that the electronic structure of MnBi_4_Te_7_ is strictly related to its magnetism, allowing for the observation of several topological states modulated by a magnetic field. Similarly, strong coupling between electronic and magnetic properties has been observed in the canted AFM state of MnBi_2_Te_4_ as a result of the net Berry curvature in the momentum space induced by the noncollinear spin structure.^47^ Compared with MnBi_2_Te_4_, the weaker interlayer exchange interactions in MnBi_4_Te_7_ have significant influences on the TH value. We observed the maximum TH value at a much lower field (∼0.5 T) for MnBi_4_Te_7_ compared to that of MnBi_2_Te_4_ (∼5 T), which has significant advantages for spintronic applications.^47^ However, the magnetic structure of MnBi_4_Te_7_ at angles around θ = 75° should be investigated further.

## 4 Conclusions

In summary, we synthesized a MnBi_4_Te_7_ single crystal and studied its magnetic topological properties. The crystal exhibits large magnetocrystalline anisotropy owing to its two-dimensional layered structure; this is supported by magnetization measurements. We systematically investigated the angle-dependent electrical transport properties and revealed an unanticipated THE in the MnBi_4_Te_7_ single crystal. A large TH resistivity of ∼7 μΩ-cm is obtained at 2 K because of the formation of a noncoplanar spin structure when the angle between the applied magnetic field and the *c*-axis is 75°. This value is significantly higher than the values for previously reported systems such as noncollinear compounds and skyrmionic and frustrated magnets. In this measurement configuration, the AFM state of MnBi_4_Te_7_ experiences a canted state from a spin-flip transition, resulting in a noncoplanar spin structure. The large THE in this system due to the noncoplanar spin configuration makes MnBi_4_Te_7_ a potential candidate for spintronic applications. Our strategy can be extended to several two-dimensional AFM topological insulator families in which the THE may exist but has not been observed till now.

## Supporting Information Available

PXRD, selected oscillation images, EDXS, isothermal magnetization, in-plane and out of plane resistance, field-dependent magnetoresistance and resistivity, Hall resistivity as a function of θ, and composition of MnBi_4_Te_7_ (Table S1) (PDF)
