# Supplementary material for: Giant Topological Hall Effect in the Noncollinear Phase of Two-Dimensional Antiferromagnetic Topological Insulator MnBi4Te7
Source: Chem Mater. 2021 Oct 19;33(21):8343–50. doi: 10.1021/acs.chemmater.1c02625 (PMC8582087; doi:10.1021/acs.chemmater.1c02625)
Supplement: Supplementary file 1 — cm1c02625_si_001.pdf [file cm1c02625_si_001.pdf]

<u>Supporting Information (SI)</u>

Giant Topological Hall Effect in Noncollinear Phase of Two-dimensional Antiferromagnetic Topological Insulator $MnBi_4Te_7$

Subhajit Roychowdhury, Sukriti Singh, Satya N. Guin, Nitesh Kumar, Tirthankar Chakraborty, Walter Schnelle, Horst Borrmann, Chandra Shekhar, and Claudia Felser**

Max Planck Institute for Chemical Physics of Solids, Dresden 01187, Germany
E-mail: Subhajit.roychowdhury@cpfs.mpg.de; Claudia.Felser@cpfs.mpg.de

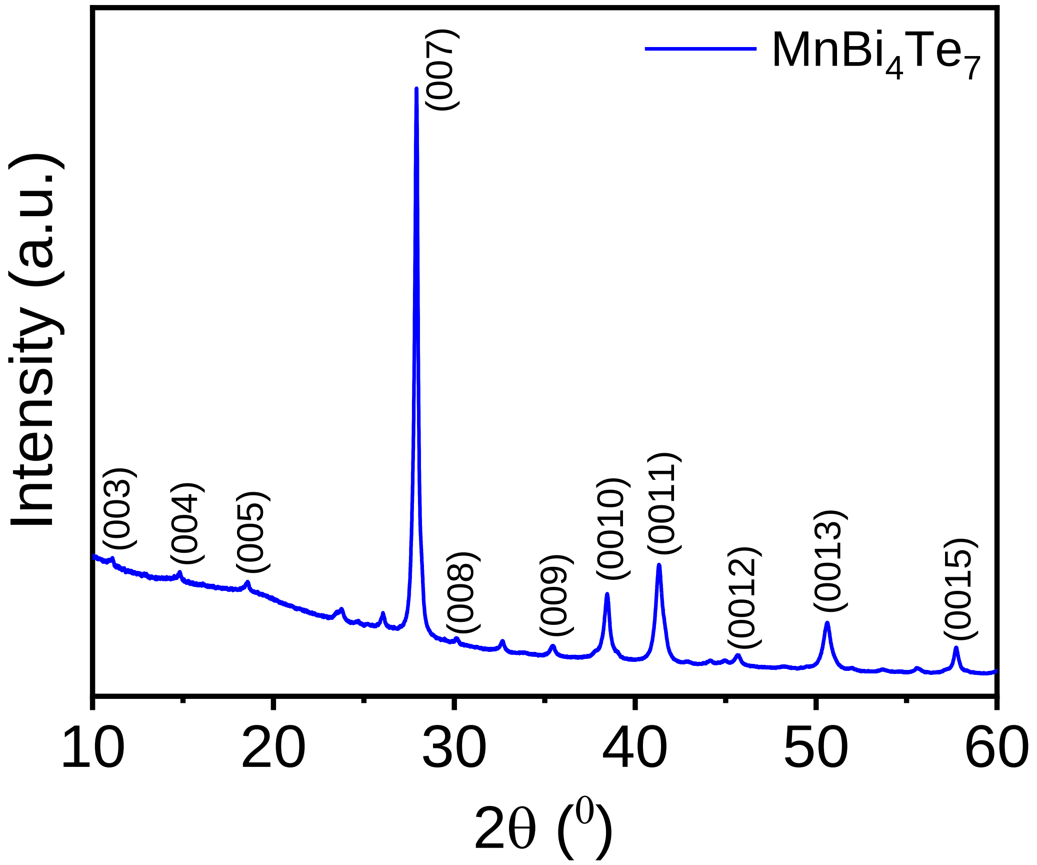

Figure S1: Powder X-ray diffraction (PXRD) pattern of the crushed $MnBi_4Te_7$ crystals (λ = 1.54056 Å).

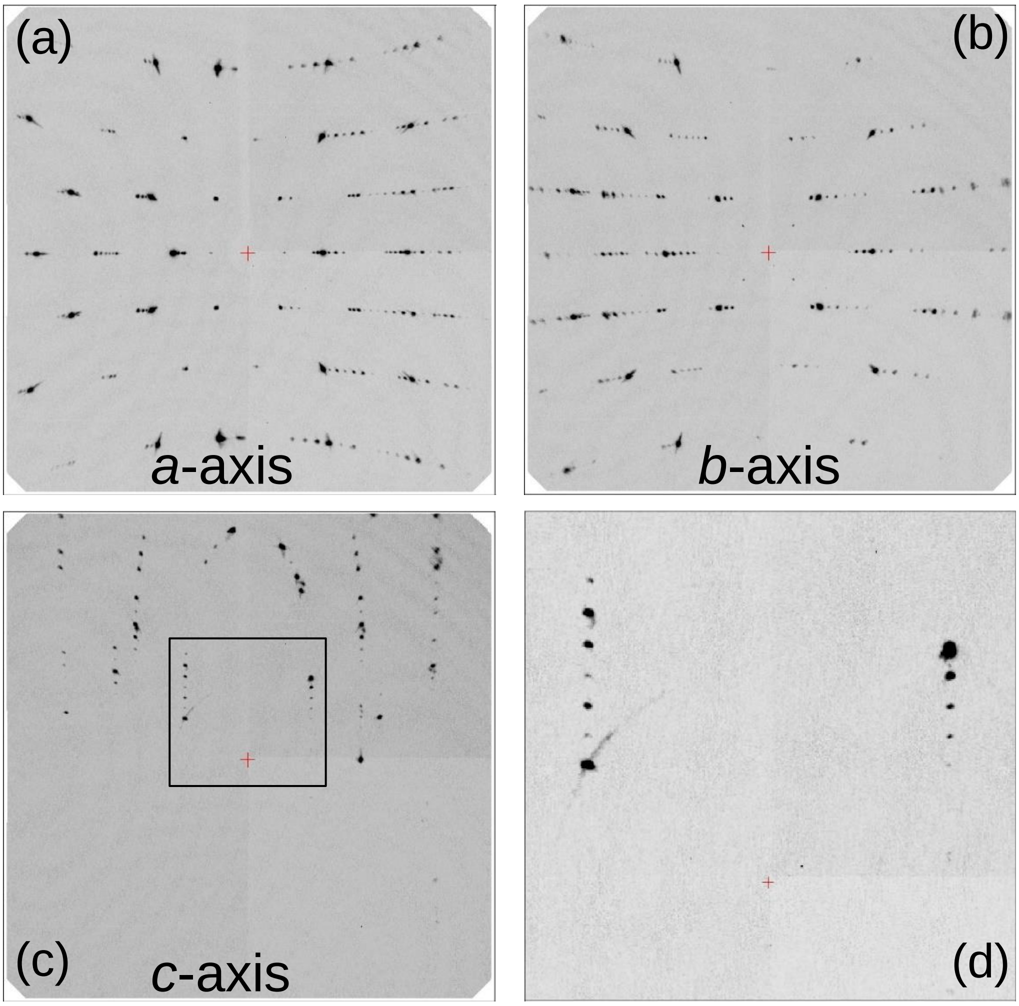

Figure S2: Selected oscillation images of $MnBi_4Te_7$ single crystals around (a) *a*-axis, (b) *b*-axis and (c) *c*-axis (on the lower part of the image no reflections are depicted due to absorption effects of the crystal). (d) shows detail within (c).

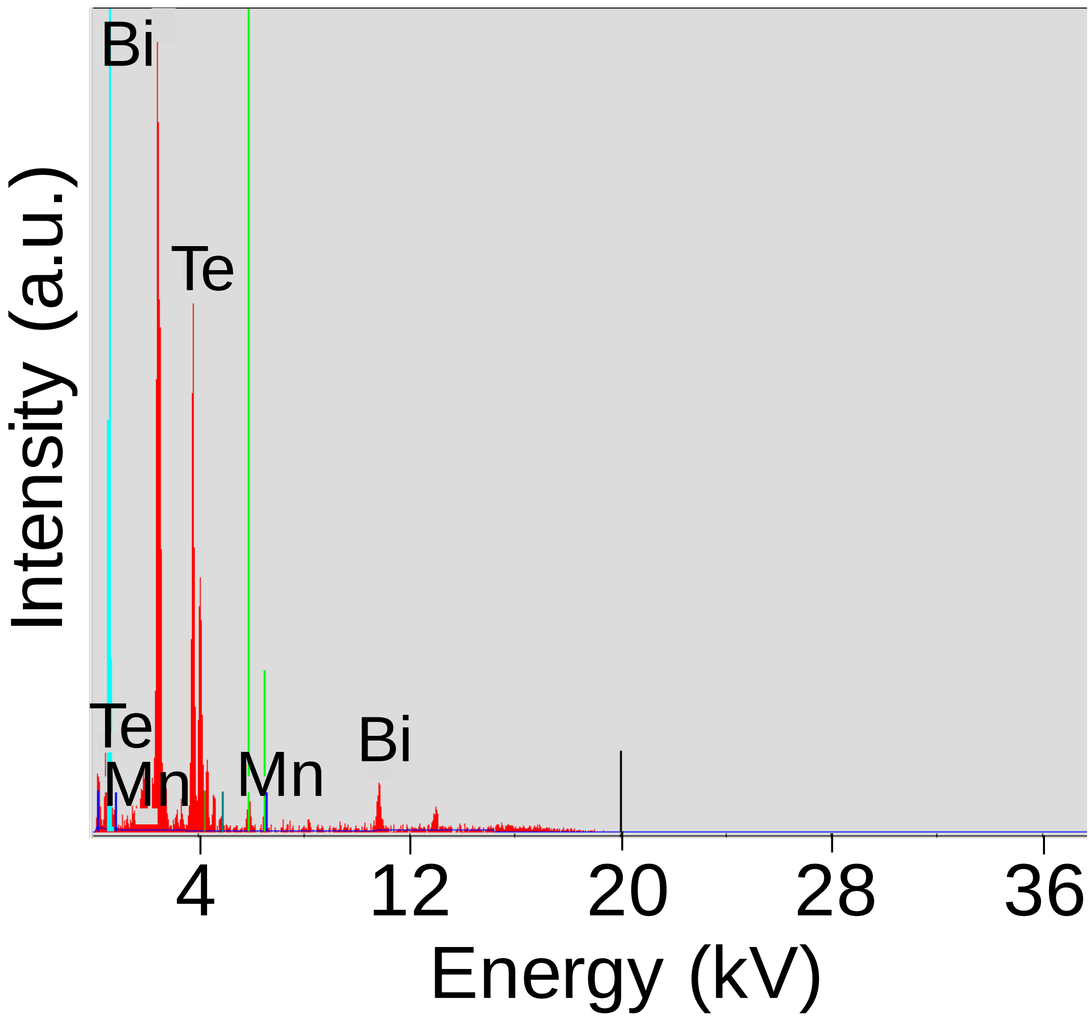

Figure S3: Energy Dispersive X-ray Spectroscopy (EDXS) spectra of $MnBi_4Te_7$ crystal.

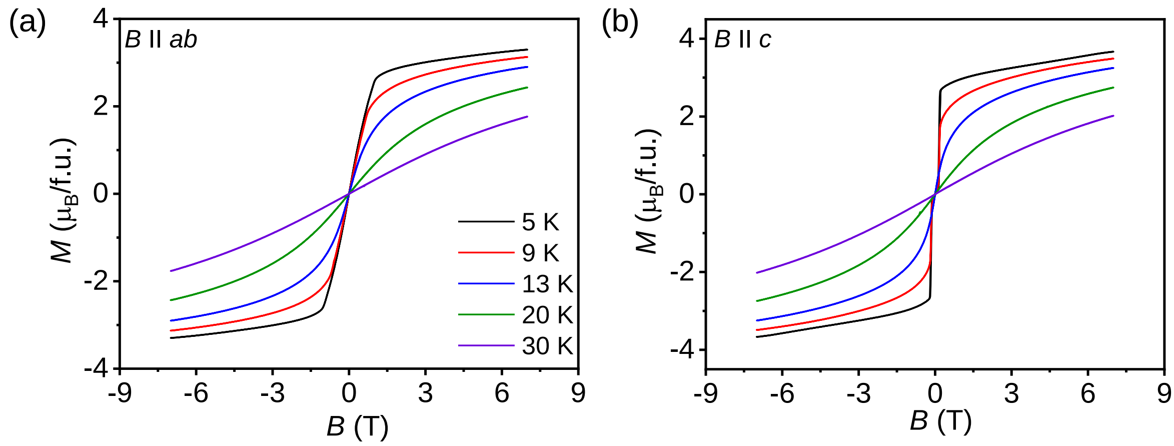

Figure S4: Isothermal magnetization for (a) *B* || *ab* and (b) *B* || *c* at different temperatures.

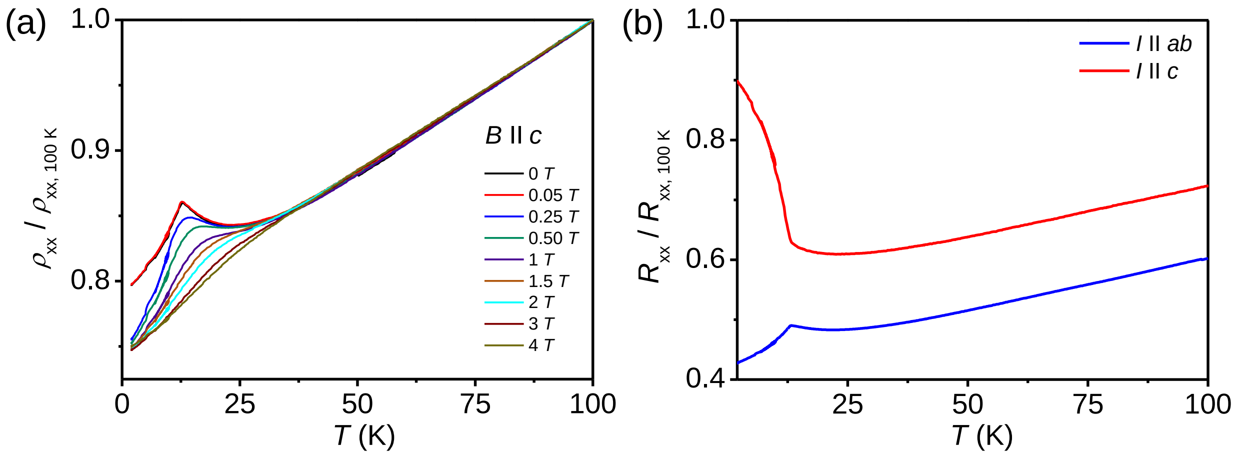

Figure S5: Temperature dependences of (a) in-plane resistivity, $\rho_{xx}/\ \rho_{xx,100\ K}$ under various magnetic fields ($B \parallel c$) and (b) $R_{xx}/\ R_{xx,100\ K}$ for I || *ab* (in-plane) and I || *c* (out of plane).

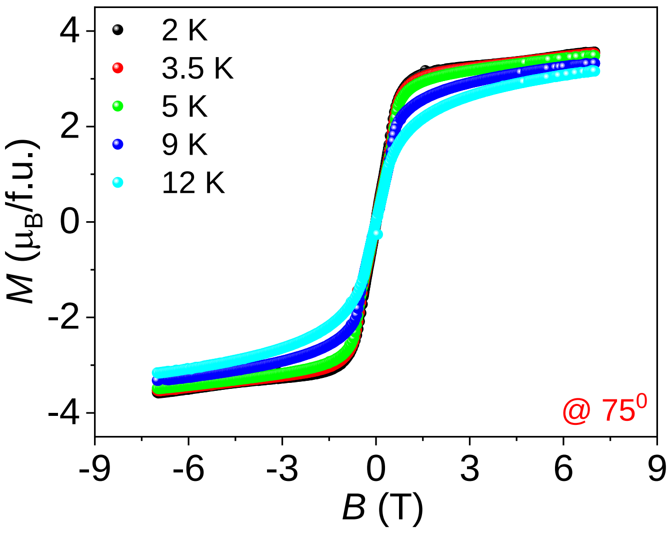

Figure S6: Isothermal magnetization for field is 75^0 from *c*-axis at different temperatures.

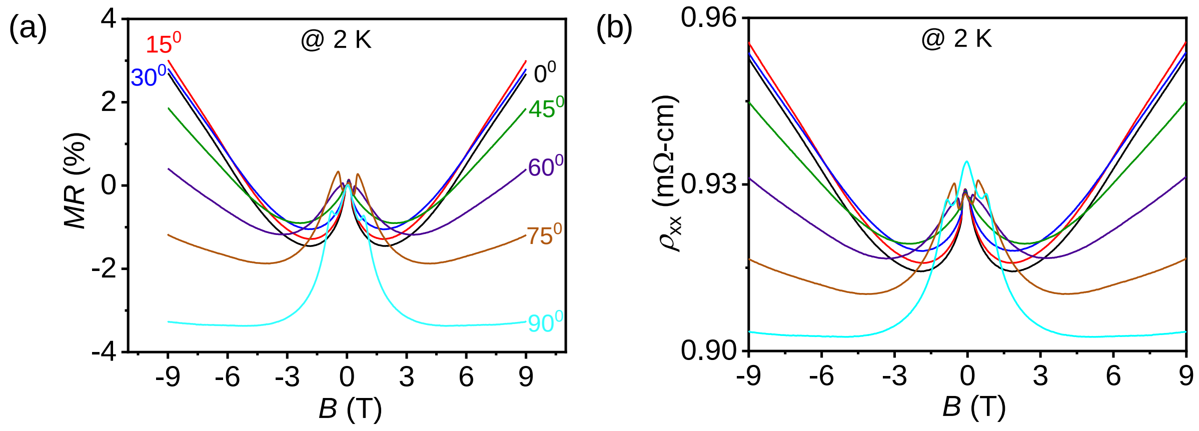

Figure S7: Field dependent (a) magnetoresistance and (b) resistivity at different θ.

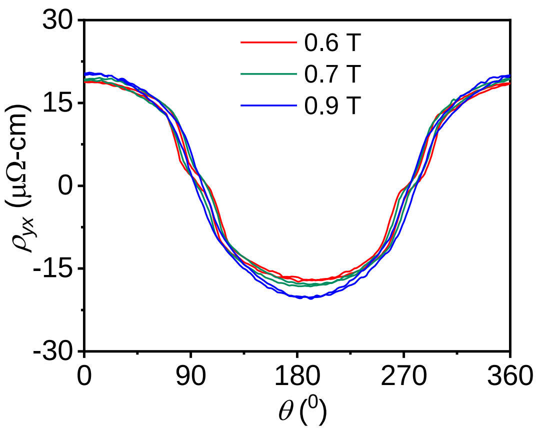

Figure S8: Hall resistivity at 3.5 K as a function of θ at different fields.

Table S1: Analysis of composition of $MnBi_4Te_7$ single crystal by Energy Dispersive X-ray Spectroscopy (EDXS):

**Nominal Composition**	**Composition obtained from EDXS**
$MnBi_4Te_7$	$Mn_{0.88}Bi_{4.5}Te_7$
